# Solid‐in‐oil nanodispersions for transdermal drug delivery systems

**DOI:** 10.1002/biot.201600081

**Published:** 2016-08-16

**Authors:** Momoko Kitaoka, Rie Wakabayashi, Noriho Kamiya, Masahiro Goto

**Affiliations:** ^1^ Graduate School of EngineeringKyushu UniversityFukuokaJapan; ^2^ Center for Transdermal Drug DeliveryKyushu UniversityFukuokaJapan; ^3^ Center for Future ChemistryKyushu UniversityFukuokaJapan

**Keywords:** Nanocarrier, Solid‐in‐oil nanodispersion, Transcutaneous immunotherapy, Transdermal drug delivery, Vaccine

## Abstract

Transdermal administration of drugs has advantages over conventional oral administration or administration using injection equipment. The route of administration reduces the opportunity for drug evacuation before systemic circulation, and enables long‐lasting drug administration at a modest body concentration. In addition, the skin is an attractive route for vaccination, because there are many immune cells in the skin. Recently, solid‐in‐oil nanodisperison (S/O) technique has demonstrated to deliver cosmetic and pharmaceutical bioactives efficiently through the skin. S/O nanodispersions are nanosized drug carriers designed to overcome the skin barrier. This review discusses the rationale for preparation of efficient and stable S/O nanodispersions, as well as application examples in cosmetic and pharmaceutical materials including vaccines. Drug administration using a patch is user‐friendly, and may improve patient compliance. The technique is a potent transcutaneous immunization method without needles.

**Abbreviations**
APC: antigen‐presenting cell
APM: L‐ascorbic acid phosphate magnesium
DC: dendritic cell
dDC: dermal dendritic cell
DFNa: diclofenac sodium
DOPE: 1,2‐dioleoyl‐sn‐glycero‐3‐phosphoethanolamine
FITC: fluorescein isothiocyanate
HLB: hydrophilic‐lipophilic balance
HRP: horseradish peroxidase
IPM: isopropyl myristate
LC: Langerhans cell
ODN: oligodeoxynucleotide
OVA: ovalbumin
PBS: phosphate buffered saline
PRR: pattern recognition receptor
s.c. injection: subcutaneous injection
S/O: solid‐in‐oil
TAA: tumor‐associated antigen
Th1 cell: type 1 helper T cell
Th2 cell: type 2 helper T cell
TLR: Toll‐like receptor
W/O: water‐in‐oil

## 1 Introduction

Transdermal drug delivery is a technique to administer drugs via the skin for systemic distribution as well as for local distributions 1, 2, 3. The method is receiving much attention as an alternative to conventional drug administration techniques. In their 2015 guidelines, the World Health Organization (WHO) recommended the oral route as the primary route of drug administration. However, the route is inadequate for drugs that are unstable under acidic conditions, or easily degraded with digestive enzymes. In addition, absorption efficiencies of large molecules through this route are low. Hence, increasing numbers of drugs, especially vaccines, are administered by injection. However, injection carries dangers of unexpected infections caused by reuse of needles and syringes, and accidental needle‐stick injuries, as well as sharp waste problems 4. According to the WHO, 21 million, 2 million and 260 000 people were newly infected with hepatitis B virus, hepatitis C virus and HIV, respectively, in the last 15 years, because of the reuse of the injection equipment. In addition, nearly 40% of the new hepatitis virus infections, and 5.5% of new HIV infections in healthcare workers were caused by accidental injuries. Transdermal drug administration using patches is anticipated to eliminate needle stick‐related problems. Moreover, the convenience of administration enables self‐administration and enhances patient compliance.

The skin has a barrier function that protects the body from invasion of environmental substances and microorganisms; therefore, skin patches use various systems to enhance physical or chemical permeation to disrupt the barrier 3, 5. Representative physical enhancers are iontophoresis 6, 7, sonophoresis 8, 9, electroporation 10, 11, and microneedles 12, 13. Chemical enhancers include some fatty acids 14, 15, terpenes 16, esters 17, alcohols 18, 19, peptides 20, 21, 22, and nanosized carriers such as lipid vesicles 23, and solid‐in‐oil (S/O) nanodispersions 24. With either type of enhancer, crossing the stratum corneum, the outermost layer in the skin, is the key to efficient transdermal drug delivery 25. In this review, we briefly describe the advantages and disadvantages of nanosized carriers including the S/O nanodispersion systems (Table 1), to overcome the skin barrier by simple application of a patch, with a focus on transcutaneous vaccination using the immune system in the skin.

**Table 1:** Brief portrayal of solid‐in‐oil nanodispersion systems

Advantages	Non‐invasive administration Slow drug‐release Easy handling Loading capacities of both hydrophilic and lipophilic adjuvants	
Disadvantages	Require homogenizer and freeze‐dryer (High‐energy formulation) Possible preference of small‐scale production	
Advantageous application area	Transcutaneous delivery of proteins and peptides (Protein drugs and vaccines)	
Drug delivery efficiencies (cumulative amounts in the skin)a)	DFNab) 72 APM 56 Insulin 70 Insulinc) 73	138.9 ± 39.4 µmcm^−2^ (46.6 ± 27.9 µm cm^−2^) 0.25 ± 0.01 µmcm^−2^ (0.12 ± 0.01 µm cm^−2^) 1.02 ± 0.26 µmcm^−2^( 0.13 ± 0.06 µm cm^−2^) 9.74 ± 4.26 µmcm^−2^ (0.25 ± 0.03 µm cm^−2^)

a) Cumulative amounts of drugs in the Yucatan micropig skins after application of the S/O nanodispersion systems for 24 h (APM) or 48 h (DFNa and insulin) were examined using Frantz‐type diffusion cells. Results show mean ± standard deviations. Controls were tested using naked drugs in IPM (DFNa) or in PBS (APM and insulin) and the results were showed in the brackets.

b) Cumulative amounts of DFNathrough the skin.

c) Surfactant L‐195 was used instead of ER‐290, and hexa‐arginine was co‐encapsulated in the nanoparticle.

## 2 Scope of transdermal drug delivery

### 2.1 Advantages over conventional oral or needle‐stick based administration methods

Since Zaffaroni marshaled advantages of transdermal drug delivery in 1981 26, the skin has been targeted as an attractive route of drug administration, and transdermal drug delivery could be an alternative to conventional oral administration and intramuscular, intradermal and subcutaneous injections. The advantages include avoidance of gastrointestinal absorption or first‐pass elimination, providing painless routine administration and enabling a steady drug concentration in the body by the slow drug‐release rate. The gradual drug influx also permits immediate cessation of treatment when adverse events occur, such as anaphylaxis during vaccine administration. Regarding vaccine administration, efficient immunization is anticipated by the transcutaneous route 27, 28, 29, 30, because a variety of immune cells are found in the skin, including Langerhans cells (LCs) residing in the epidermis, and dermal dendritic cell (dDC) subsets in the dermis 31. However, the stratum corneum, the outermost layer in the skin, becomes a physical barrier when a drug permeates the skin.

### 2.2 Structure of the skin

The skin consists of three stratiform tissues, epidermis, dermis and subcutaneous tissue. The epidermis comprises the stratum corneum and viable epidermis. Viable epidermis and dermis are hydrophilic and the stratum corneum is hydrophobic. The stratum corneum is 15 µm thick and consists of 10–20 layers of corneocytes buried in lipids such as ceramides, cholesterol esters and fatty acids 32, 33, 34. There is a little amount of water in the stratum corneum (10–25%), which is located between the lipid bilayers to form lamellar phases 35, 36. The average thickness of the extracellular space between the corneocytes is 44 nm, which corresponds to eight lines of lipid bilayers 37. Corneocytes are filled with keratin, unlike other cells. The hydrophobic and fibrous structure of the stratum corneum prevents dehydration of the body and invasion of exogenous materials. In addition, adhesive membrane proteins form tight junctions in viable epidermis, and act as the second physical barrier. Accordingly, molecules with modest lipophilicity and small molecular weight are likely to permeate through the skin by passive delivery, which is known as the 500 Dalton rule 38, 39, 40. Chemical permeation enhancers such as nanosized carriers facilitate drug permeation through the stratum corneum toward blood vessels. Regarding the permeation of small to large solutes through the stratum corneum, four different pathways are proposed: free‐volume diffusions and lateral lipid diffusions for hydrophobic solutes, and the pathways through shunts and pores for hydrophilic solutes 41.

### 2.3 Deliveries of peptide/protein drugs

Recently, proteins and peptides have been being exploited for medical use to treat various diseases and immune disorders 42. These include insulin, human growth hormones, and peptide vaccines for cancers and allergies. The bioactive protein/peptide drugs are hydrophilic and often charged, with molecular weights ranging from 1 to > 50 kDa. Permeation efficiencies of intact proteins and peptides through the stratum corneum are low; therefore, many studies have investigated delivering these biomolecules efficiently into the viable epidermis and dermis.

The slow permeation efficiency is favorable for deliveries of whole antigen molecules such as vaccines for immunotherapy of allergic diseases, because it may reduce the vital risk during therapy. Epicutaneous immunotherapy (EPIT) using a patch loaded with whole milk by electrospray deposition has succeeded in inducing clinical tolerance in patients with cow's milk allergy by delivering the antigen slowly to the epidermis 43. It is noteworthy that no serious adverse event was reported during the EPIT trials. Although several patients showed local erythema/eczema at the position of the patch, the treatment discontinuation rate was low compared with that of oral and sublingual immunotherapy, indicating the potential application of cutaneous immunotherapy. EPIT is now undergoing phase 3 in clinical trials. In contrast, immunotherapy using whole antigen molecules requires a long period of treatment. Therefore, the use of T cell epitope peptides is being explored to increase the dose and reduce the treatment duration.

## 3 Nanocarriers for transcutaneous protein/peptide delivery

Nanocarriers have been extensively investigated for convenient and noninvasive protein/peptide delivery across the skin 44. Nanocarriers for transdermal drug delivery are divided in two categories: those that are dispersed in hydrophilic vehicles or in lipophilic vehicles. Hydrophilic lipid vesicles are pioneering nanocarriers that efficiently deliver proteins/peptides to the skin. Cevc et al. have developed Transfersomes, which are deformable and squeeze into the stratum corneum through the intercellular and intracellular pathways, and demonstrated the clinical effectiveness of the carrier by the transdermal route 45. Elastic liposomes and ethosomes have been applied to immunotherapy, and these carriers elicit high levels of antibodies comparable to those with injection 46, 47, 48. Biodegradable and biocompatible polymers also serve as hydrophilic drug carriers. Poly(lactide‐co‐glycolic acid) nanoparticles are prepared and modified with ease 49. Chitosan is a cationic polysaccharide; thus, the carrier itself has potential as a skin permeation enhancer that interacts with the negatively charged cell surface 50, 51. Combinations of the above mentioned carriers and skin‐permeation‐enhancing peptides or physical enhancing methods have been used in recent research for an enhanced transdermal drug delivery.

On the contrary, nanocarriers dispersed in lipophilic vehicles are supposed to penetrate the stratum corneum efficiently, because the stratum is hydrophobic. Oil‐based nanocarriers include water‐in‐oil (W/O) microemulsions, also referred to as nanoemulsions, solid lipid nanoparticles and S/O dispersions 52, 53, 54, 55. Typical mean particle sizes of W/O micro emulsions are < 500 nm. Compared with other emulsions, microemulsions are thermodynamically more stable as a result of a decreased particle size and a smaller surface tension between the oil and water phases. S/O nanodispersions are also < 500 nm in size. They are oil‐based dispersions of solid powders of hydrophilic molecules. S/O dispersions are prepared by removal of water and cyclohexane from W/O emulsions by lyophilization, and redispersion of the surfactant–drug complex in another oil vehicle. Preferably, the oil vehicle has a skin‐penetration‐enhancing property 24. Advantages of S/O nanodispersions over W/O microemulsions are drug loading capacity and stability. S/O particles are filled with drug compounds, while droplets of W/O microemulsions contain large amounts of water molecules, which reduces the drug concentration in the final products. Encapsulation efficiency of the S/O particles is high. Okuma et al. reported that 80% of l‐ascorbic acid phosphate magnesium (APM) was encapsulated inside the surfactant of S/O nanocarriers 56. Similar high encapsulation efficiencies were also reported when bovine serum albumin (66 kDa) and various molecular weights of hyaluronic acids were enclosed into the S/O nanoparticles 57. S/O dispersions are stable for more than three months, because of fewer opportunities of Ostwald ripening 58, 59. Particles tend to aggregate after several months of storage; however, they are easily redispersed by vortex agitation or ultrasonication. Removal of water molecules reduces the opportunities for hydrolysis of internal molecules 60.

## 4 Development of the S/O nanodispersions

### 4.1 Selection and formulation of surfactants

**Figure 1:**
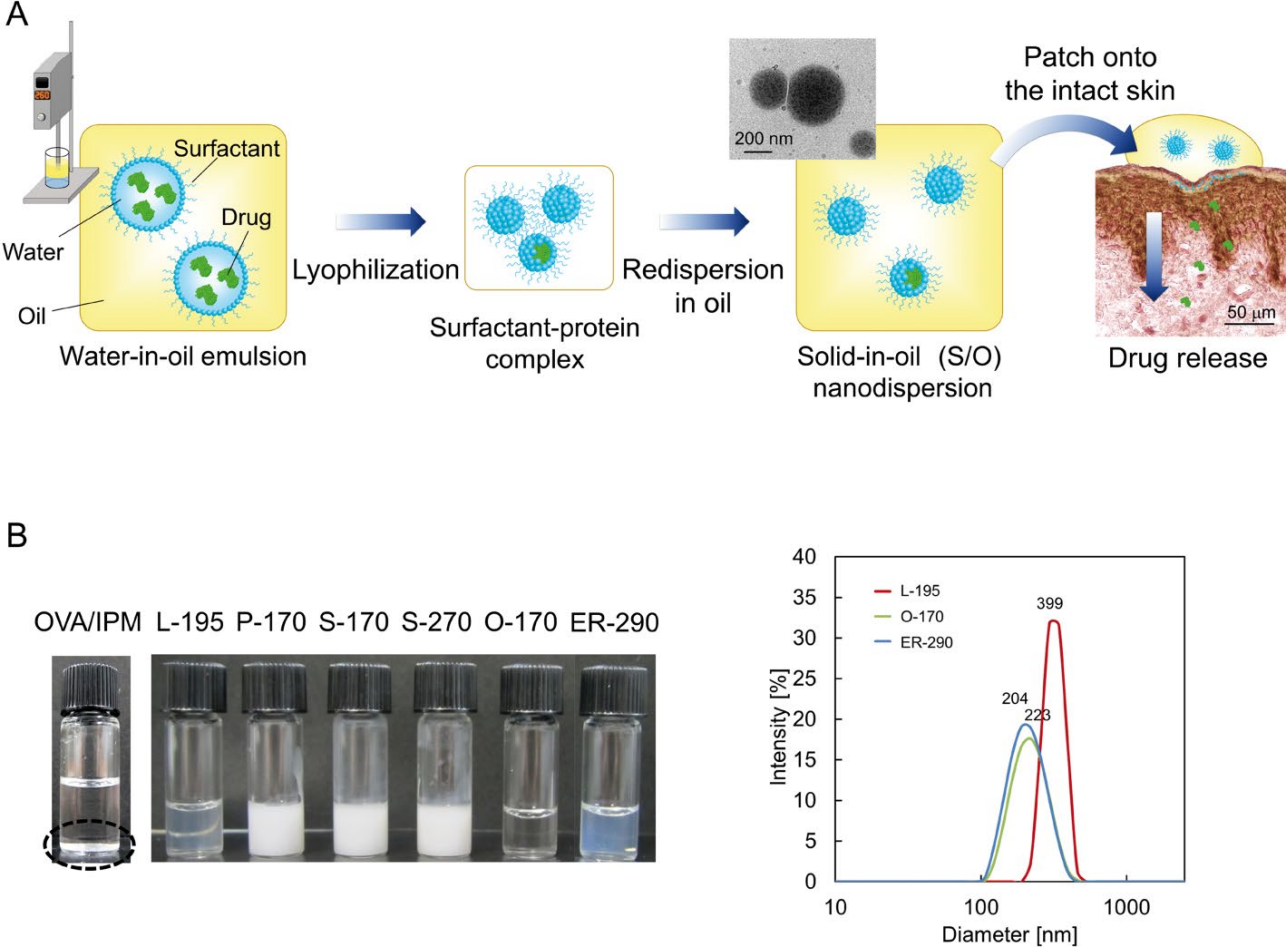
Preparation and application methods (**A**) and physical appearances and size distribution curves (**B**) of S/O dispersions. W/O emulsions prepared by ultrasound, rotor‐stator or high pressure homogenization are subjected to lyophilization, and then the surfactant/drug complex is redispersed in oilvehicles to form S/O nanodispersions. The nanodispersions are applied to the intact skin using patches. Sucrose laurate (L‐195), sucrose palmitate (P‐170), sucrose stearate with HLB 1 and 2 (S‐170 and S‐270, respectively), sucrose oleate (O‐170), and sucrose erucate (ER‐290) were examined to prepare S/O nanodispersions containing OVA, and L‐195, O‐170 and ER‐290 formed stable nanosized dispersions, while naked OVA was insoluble in IPM. Particle size distribution was measured by dynamic light scattering using Zetasizer NanoZS (Malvern). Reproduced with permission 66. Coypright 2014, Royal Society of Chemistry.

The S/O nanodispersion system is composed of hydrophilic drug particles dispersed in an oil vehicle by the assistance of hydrophobic surfactants (Fig. 1A). The drug delivery efficiency across the skin is influenced by its formulation (i.e. concentration and type of surfactants, oil vehicles, and surfactant–drug ratios). Isopropyl myristate (IPM) is the preferred oil vehicle for S/O nanodispersions 61, 62, 63, because it enhances drug permeation through the stratum corneum because of its ability to interact with lipids in the stratum corneum. Moreover, IPM is safe and widely used in cosmetic and pharmaceutical products. W/O emulsions, precursors of the S/O nanoparticles, are prepared by homogenizing bioactive molecules in water and lipophilic surfactants in a volatile oil. Cyclohexane is suitable as the volatile oil because its freezing point of 6.5°C is close to that of water, allowing the water and oil phases to be frozen simultaneously in liquid nitrogen. In addition, the high vapor pressure of cyclohexane permits immediate evanescence up only ophilization. Chloroform is also applicable for preparation of W/O emulsions prior to lyophilization 57, 59. High pressure homogenization and an ultrasound method are also applicable for the formation of stable W/O emulsion precursors 64, 65. The role of these techniques is comprehensively reviewed elsewhere 65.

A series of sucrose fatty acid esters are often used as surfactants comprising S/O nanoparticles. In foods, cosmetics and pharmaceuticals, sucrose fatty acid esters are widely used as an emulsifier. Their hydrophilic/lipophilic balance (HLB) varies from 1 to 18 depending on the carbon chain length and degrees of unsaturation in fatty acids and esterification. Surfactants with HLB 1–3 are preferable for preparation of S/O nanoparticles. In a previous report, a series of 25 mg/mL sucrose fatty acid esters (HLB 1–3) in a cyclohexane solution were examined to prepare S/O nanodispersions 66. As a result, three surfactants, sucrose laurate, sucrose oleate and sucrose erucate formed stable S/O nanodispersions (Fig. 1B). Surfactants composed of saturated fatty acids of > 16 carbon chain length did not dissolve in cyclohexane at 25 °C. Consequently, they did not form nanosized dispersions. According to other reports, sucrose stearate in chloroform can be used for S/O nanodispersion 57, 59.

The particle size and drug release efficiency are influenced by the drug to surfactant ratio. The surfactants composed of longer chains of fatty acids formed smaller and more stable nanoparticles 66. Similar to emulsion systems, the particle size decreases along with an increase of the drug/surfactant ratio 66, 67, 68. Larger particles are likely to disintegrate and release drugs more efficiently. Therefore, the S/O nanodispersions composed of smaller drug‐surfactant ratios induced higher antigen‐specific antibody responses 66. Encapsulation of strong polyelectrolytes facilitates particle disintegration, while other polymers such as hyaluronic acid are used to reinforce the particle stability 59, 69.

### 4.2 Drug delivery pathways with the S/O nanodispersion system

**Figure 2:**
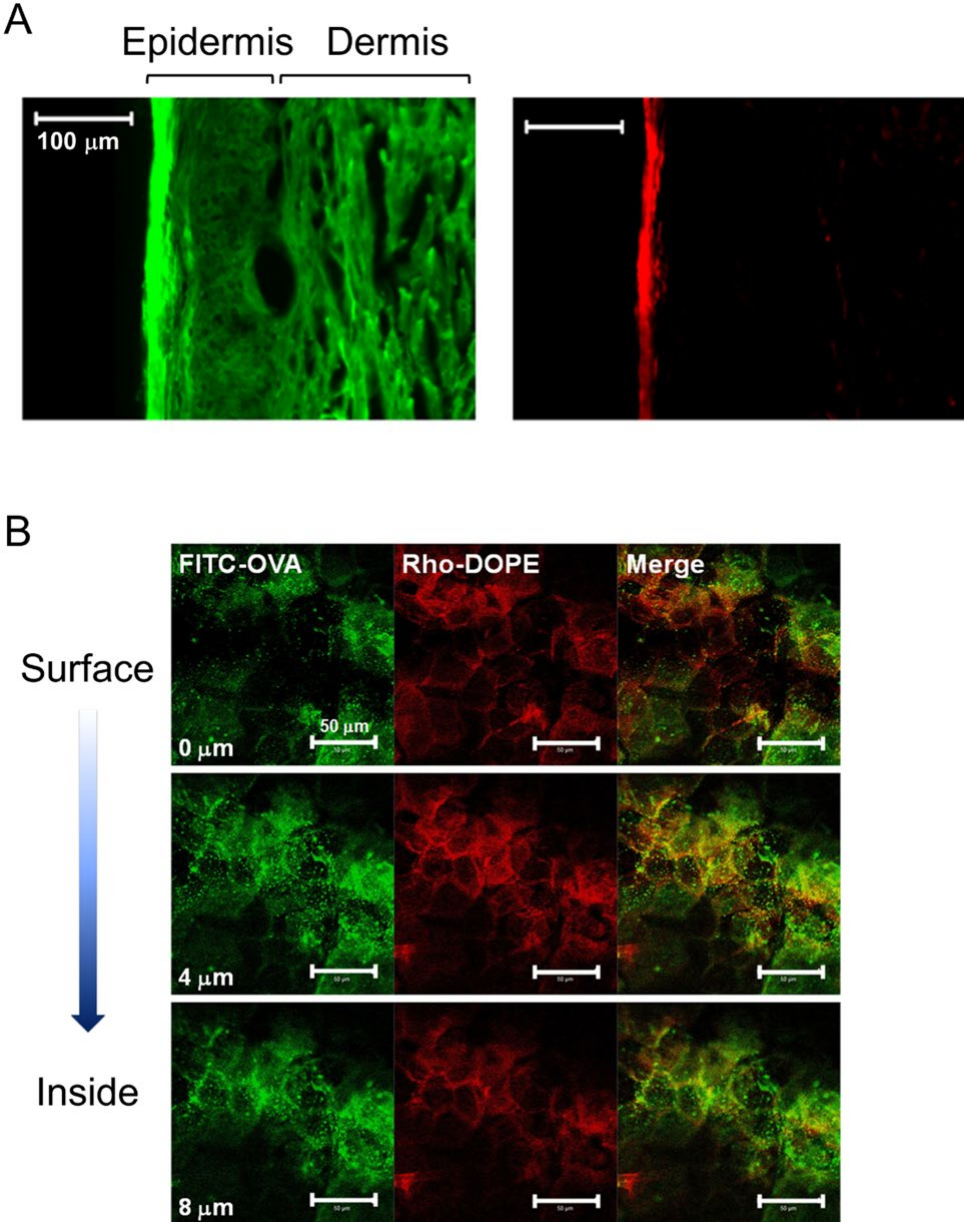
Fluorescence microscopic images of Yucatan micropig skin sections (**A**), and mouse ear epidermal sheet (**B**), adapted with permission 66. Coypright 2014, Royal Society of Chemistry and adapted with permission 70. Coypright 2008, Elsevier, respectively. The Yucatan micropig skin was treated with S/O nanodispersions composed of fluorescein isothiocyanate (FITC)‐labeled insulin and rhodamine‐labeled 1,2‐dioleoyl‐sn‐glycero‐3‐phosphoethanolamine (rhodamine‐) for 48 h, and sectioned for observation by fluorescence microscopy. Another S/O nanodispersion consisting of FITC‐labeled OVA and rhodamine‐DOPE was applied to mouse ear for 24 h, and then the epidermal sheet was isolated from the ear and observed by confocal laser scanning fluorescence microscopy. Rhodamine‐DOPE mostly remained in the stratum corneum, whereas, FITC‐protein permeated the viable epidermis and dermis. The lipid and proteins penetrated the intercellular pathways.

There are three pathways for organic nanoparticles to permeate the stratum corneum: lateral diffusion along the corneocytes; permeation across corneocytes; and routes through follicles and glands 44. Besides, it is known that hydrophilic molecules do not permeate the stratum corneum, while hydrophobic molecules remain in the stratum corneum. How does the S/O nanodispersion system deliver hydrophilic compounds through the skin? Previous studies of S/O nanodispersions revealed that the hydrophobic surfactants are likely to be removed from the drugs in the stratum corneum, and only hydrophilic molecules infiltrate the viable epidermis and dermis (Fig. 2A). Computational dynamics simulation also indicated rapid dissociation of surfactants by contact of S/O particles with the lipid membrane 59, 71. The components of particles were supposed to be integrated immediately into the lipid bilayers. Confocal microscopic images clarified that the S/O nanoparticles were delivered by intercellular routes (Fig. 2B). These results indicate that the S/O nanodispersion system is highly recommended for delivery of hydrophilic molecules such as proteins and peptides.

## 5 Application of S/O nanodispersion systems

### 5.1 Transdermal drug delivery

Piao et al. first adapted the S/O nanodispersion system to transdermal drug delivery 72. They prepared small S/O particles (average particle size ≈15 nm) containing diclofenac sodium (DFNa; Mw 296), an anti‐inflammatory drug. DFNa in S/O nanoparticles showed enhanced skin permeability compared with that of the aqueous naked DFNa solution. Subsequently, the S/O nanodispersion system was applied to the topical delivery of cosmetic ingredients of small to large molecular weight, such as APM 58, hyaluronic acids and protease inhibitor peptide 71. Accumulating amounts of APM and hyaluronic acid in the skin increased by three‐ and four‐fold, respectively, by encapsulating in the S/O particles.

The S/O nanodispersion system is also applicable to the transcutaneous deliveries of protein drugs and enzymes, without losing their conformations or enzymatic activities. Tahara et al. demonstrated the delivery of fluorescein‐isothiocyanate‐labeled insulin (6 kDa) using the Yucatan micropig skin 70. The fluorescence images of skin sections revealed that insulin permeated the skin as incubation time increased. On the contrary, insulin in phosphate‐buffered saline (PBS) hardly permeated the skin. The effects of the S/O system on enzymatic activities in and out of nanoparticles were also assessed using horseradish peroxidase (HRP; 40 kDa). The skin subjected to HRP‐containing S/O nanodispersion was sectioned and incubated with HRP substrate, metal‐enhanced diaminobenzidine, and H_2_O_2_, and then the substrate‐specific brown color developed in the epidermis. Besides, HRP and lysozyme maintained > 90% of enzymatic activities in the S/O particles for 24 h, showing a high potency of the S/O nanodispersion as a protein carrier for the transdermal drug delivery 68. The transdermal delivery of insulin was improved by co‐encapsulation of oligoarginines 73. Recent research has revealed that there are tight junctions between epithelial cells that act as the second physical barrier to protect from infiltration of pathogens and endotoxins 74, 75. Denaturation of the membrane proteins comprising the tight junctions by cation‐rich peptides increases protein penetration into the skin.

### 5.2 Preventive vaccination

Vaccines nowadays are used to prevent infectious diseases and to cure antigen‐related diseases. Preventive inoculation has reduced the prevalence of serious diseases caused by viruses and other pathogens. Classical vaccination uses injection to administer vaccines directly into subcutaneous fat or muscle. However, recent studies have revealed that vaccination by the intradermal route is more effective, because numerous populations of immune cells are found more in the epidermis and dermis than in the subcutaneous tissue 76, 77. The immune‐related cells in the skin include LCs, dendritic epidermal γδ T cells and CD8^+^ tissue‐resident memory T cells in the epidermis, as well as dDC subsets, dermal γδ T cells, mast cells and CD4^+^ tissue‐resident memory T cells in the dermis 78, 79. Regulatory T cells are also found in the mouse dermis 80, 81. LCs are representative antigen‐presenting cells (APCs) in the epidermis that were discovered by Langerhans in 1868 82. Silberberg et al. reported the antigen‐presenting ability of LCs 83, and the concept of transcutaneous immunization using LCs was proposed by Glenn et al. 84. LCs can migrate to the skin‐draining lymph nodes where they present antigens to CD4^+^ and CD8^+^ T cells, or pass antigens to other DCs 85, 86, 87. Migratory DCs and circulating T effector memory cells are recruited into the dermis from blood vessels, and exit from lymphatic vessels in response to cytokine signals. Therefore, utilizing the immune cells, the skin is potent to induce strong systemic adaptive immune responses 77.

**Figure 3:**
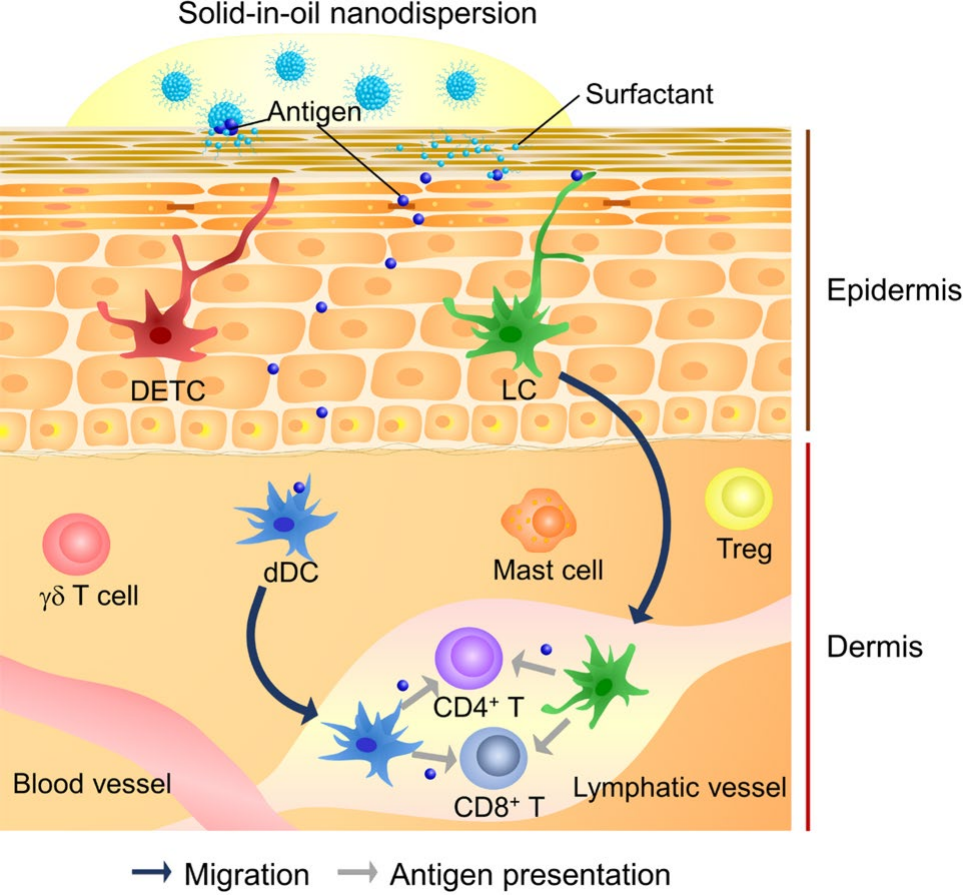
Concept of transcutaneous immunotherapy using S/O nanodispersions. Antigens are released in the stratum corneum and permeate the epidermis and dermis. LCs and dDCs capture antigen and migrate to lymphatic vessels and skin‐draining lymph nodes. They present antigens to nearby CD4+ and CD8+ T cells, or pass them to other DCs. LCs may activate T regulatory cells under steady‐state conditions by the existence of antigen, thereby inducing alleviation of allergic responses. Dendritic epidermal γδ T cells may promote Th2 cell induction, and dermal γδ T cells may induce CD4+ T cells under inflammatory conditions.

Using the S/O nanodispersion system, an antigen model protein, ovalbumin (OVA; 45 kDa) strongly induced antigen‐specific antibody responses in murine sera, even though patches loaded with S/O nanodispersions were simply applied to intact skin 66, 88. In addition, the percentage of Langerin^+^ cells that captured OVA in the skin‐draining lymph nodes doubled following encapsulation of OVA in the S/O nanodispersions 66. This indicates that OVA arrived in the Langerin^+^ cells, and then the cells migrated to the lymph nodes after capture of OVA (Fig. 3). Importantly, the OVA‐specific IgG titer correlated with the in vitro OVA release efficiency from the nanoparticles. The inner layers in the skin are hydrophilic; therefore, hydrophilic proteins leaving the nanoparticles are more likely to interact with LCs and dDC since they bypassed the stratum corneum.

OVA was more efficiently delivered to APCs when one of the skin‐penetrating peptides, hexa‐arginine, was co‐encapsulated in the S/O nanoparticles 69. However, how to repair the reduced barrier function of the skin and protect the body from infections is still controversial, especially after repeated drug administration.

Additional use of adjuvants is a promising approach for enhanced transcutaneous vaccination through activation of APCs 90, 91. Some conserved moieties in pathogens or damaged cells are recognized by pattern recognition receptors (PRRs) in an innate immune system, and activate APCs 92, 93. Under inflammatory conditions (existence of adjuvants), the activated APCs exhibit increased antigen presentation and cytokine production, although the signaling pathways vary by different PRRs and DCs 93, 94. It is also known that type 1 helper T (Th1) cells are activated by the activated B cells. Toll‐like receptors (TLRs), a family member of PRRs, and their ligands are particularly well‐studied systems because similar TLRs are expressed in humans and animal models. In a previous study using the S/O nanodispersion system, coadministration of CpG oligodeoxynucleotides (ODNs), known as ligands of TLR9 95, increased antigen‐specific antibody titer in mouse sera by nine times, and enhanced the Th1‐biased immune response 96. The CpG ODNs were supposed to be delivered to the skin‐draining lymph nodes efficiently enough to activate B cells 97. The results mentioned above indicate that the S/O nanodispersion system has a potential use in transcutaneous preventive vaccination.

Since the continuous phase of the S/O nanodispersions is IPM, additional use of lipophilic adjuvants, such as, TLR4 agonists monophosphory lipid A 98, 99, 100, trehalose‐6,6'‐dibehenate 101, 102, and lipophilic derivatives of muramyl dipeptide 103, would improve the stabilization of the S/O nanodispersions, instead of merely induction of the enhanced antibody responses. Above mentioned adjuvants are also known to induce Th1‐type immunity, which is a prerequisite for protection against tuberculosis, malaria and HIV infections.

### 5.3 Pollinosis immunotherapy

Allergen immunotherapy is the only curative therapeutic method for allergic diseases 104, 105. However, all the licensed allergen immunotherapies use whole allergen molecules that have a risk of IgE‐mediated serious adverse events. Recently, short peptides derived from allergen molecules that are recognized by T cells, known as T‐cell epitopes, were exploited to reduce adverse events during therapy 106, 107, 108. Cautiously chosen T‐cell epitopes lack the ability to bind to IgE on mast cells; therefore, use of T‐cell epitopes as vaccines is a safer strategy than the use of whole allergen molecules. Mast cells migrate into the dermis; thus, T‐cell epitope vaccines are also suitable for immunotherapy using the transcutaneous route.

Previously, seven different T‐cell epitope determinants were picked up for immunotherapy of Japanese cedar (*Cryptomeria japonica*) pollinosis that were estimated to be recognized by the T cells from 90% of the patients 109. The long polypeptides that comprised seven different peptides were expressed in rice seeds 110, or eggs 111, and the edible vaccines successfully reduced allergic symptoms in mouse models of Japanese cedar pollinosis. Edible vaccines are easy to take; however, issues arise when patients are under dietary restriction or have gastrointestinal disorders. For transcutaneous administration, a polypeptide comprised of seven T‐cell epitopes, namely 7CrpR, was produced by an *Escherichia coli* system, and encapsulated in S/O nanodispersions. Patches loaded with S/O nanodispersions containing 7CrpR were applied to a pollinosis model in mice 112. The antigen‐specific IgE level declined in sera, indicating that the peptide administered using the S/O nanodispersion system elicited a change in the immune milieu. On the contrary, 7CrpR in a PBS solution did not reduce the antigen‐specific IgE level. The skin sections that were subjected to the S/O nanodispersion containing fluorescence‐labeled 7CrpR showed that the peptide mostly remained in the stratum corneum. However, thickening of the stratum corneum was observed, indicating that the stratum corneum acted as a repository of the S/O nanoparticles. In this context, the peptide was assumed to be discharged slightly but constantly even after removal of the patches.

Recent research has revealed that the skin contains a variety of cells that are involved in the immune system. LCs are known for presentation of major histocompatibility complex (MHC) class II, activation of CD4^+^ T and T regulatory cells 113, 114, LCs in the epidermis are supposed to play an important role in cutaneous immunotherapy of pollinosis, similarly to EPIT using whole allergen molecules 43.

### 5.4 Cancer immunotherapy

Carcinogenesis is related to the immune system and therefore immunotherapy for cancer has been studied for over a century. In particular, cancer vaccines that activate the adaptive immune system against cancer are useful in that they are less likely to cause side effects such as autoimmune disorders 115. Recent development in the SEREX technology has identified several tumor‐associated antigens (TAAs) that can be used in cancer vaccines for clinical application 116, 117. Therapeutic immune response is initiated by the capture of these TAAs by DCs, followed by presentation as MHC/TAA conjugates. The presentation of antigens on MHC class I molecules called cross‐presentation is an important step for induction of cancer immunity, because once the MHC class I‐antigen conjugate is recognized by CD8^+^ T cells, the CD8^+^ T cells differentiate into cytotoxic T lymphocytes that play a major role in antitumor activity 118, 119. Epidermal LCs and Langerin^+^ dDCs are known to have high ability of cross‐presentation 120, 121, 122. Therefore, utilizing the skin immune system for cancer vaccine seems a promising approach. As with other transcutaneous immunization systems, the delivery of TAAs to DCs in the skin is the key to efficient cancer immunotherapy. Seo et al. reported the first percutaneous cancer vaccine by topical application of TAAs on the stratum corneum in the barrier‐disrupted skin using a tape‐stripping method 123. Besides this tape‐stripping method 124, 125, 126, other skin‐permeation‐enhancing systems, such as electroporation 127, microneedles 128, 129, and nanocarriers 130 have been demonstrated to activate the tumor‐specific immune system.

**Figure 4:**
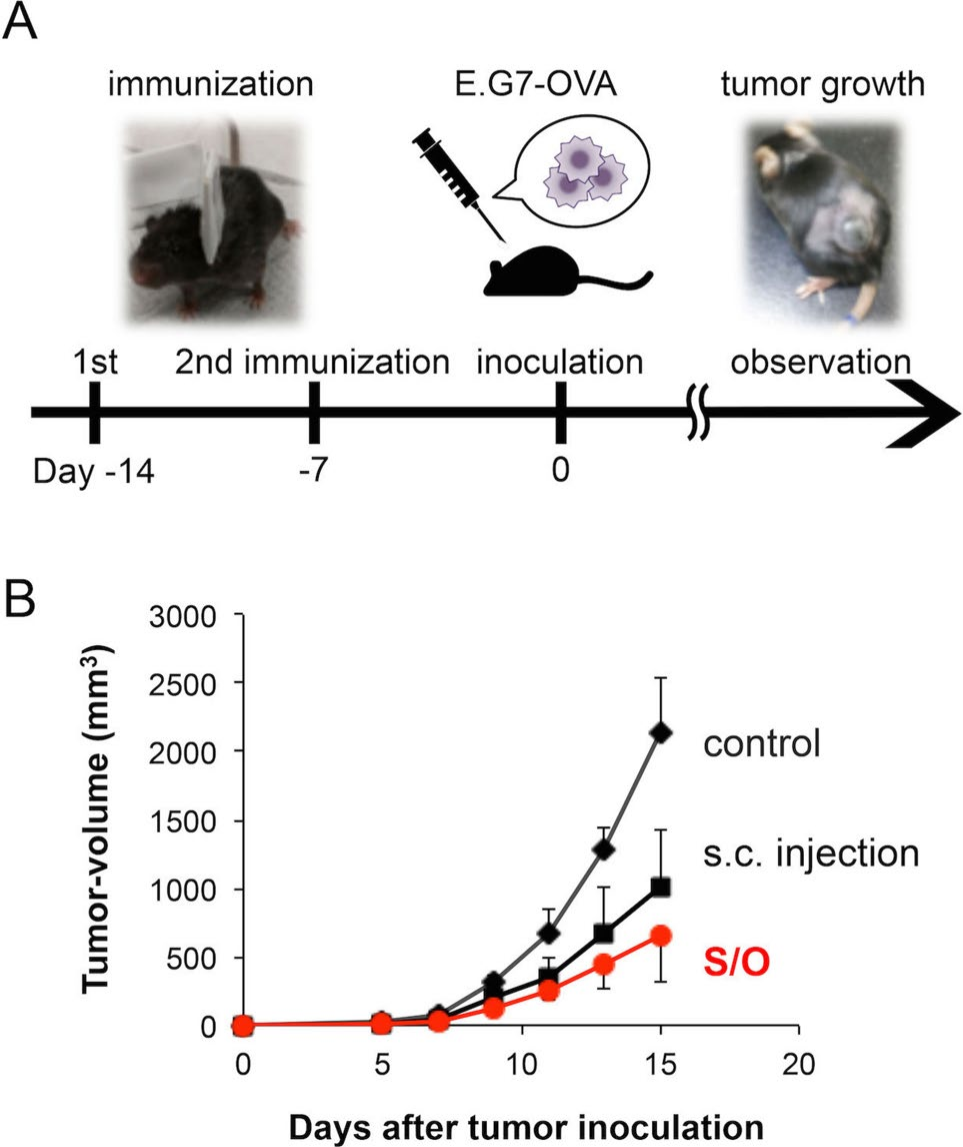
Time schedule of immunization and tumor cell inoculation (**A**), and antitumor effect of OVA administered by topical patch or s.c. injection (**B**). OVA in the S/O nanodispersion system induced antigen‐specific cancer immunity comparable to that in a PBS solution administered by s.c. injection. Adapted with permission 131. Coypright 2015, Royal Society of Chemistry.

The S/O nanodispersion system was applied to induce cancer immunity using OVA as a model cancer antigen 131. The growth inhibition of E.G7‐OVA cells, OVA expressing lymphoma, was observed in the mice vaccinated with topical application of the OVA‐loaded S/O nanodispersion (Fig. 4). It was noted that the tumor suppression level was equal to or higher than in mice immunized by subcutaneous (s.c.) injection. In cancer vaccines, CD4^+^ T‐cell‐derived Th1 cells are also important because they help to activate cytotoxic T lymphocytes by cytokine production 132. Th1 cytokine interferon‐γ is produced more from splenocytes of S/O‐treated mice than those subjected to s.c. injection, and Th2 cytokine interleukin‐4 was detected only from the splenocytes of s.c. injected mice. These results suggest that S/O nanodispersion is an efficient nanocarrier for cancer vaccine, with the ability to induce Th1‐biased immunity.

## 6 Conclusions

The transdermal drug delivery system is a promising alternative to conventional drug administration methods. Chemical skin permeation enhancers have a benefit of simple and easy delivery of proteins and peptides across the skin barrier. Moreover, vaccine administration by the cutaneous route may allow efficient immunization with a small dose, by utilizing the immune system in the skin. The S/O nanodispersion system has a potency for both transdermal drug delivery and transcutaneous immunotherapy. For enhanced drug delivery across the skin, the drug release efficiency from the S/O nanodispersion of the particles had a strong effect, and the propensity to particle disintegration may be affected by the particle size and excipients. Co‐administration of ligands that bind to acceptors inside and outside the APCs may improve the antibody response using the S/O system. The S/O system is composed of biocompatible materials, and has the potential to attain the advantages of transdermal drug delivery.
